# High-Risk Cardiomyopathy Genotypes and Arrhythmic Risk: LMNA, FLNC, RBM20, PLN and Desmosomal Genes in the ESC 2023 Era

**DOI:** 10.3390/genes17040370

**Published:** 2026-03-25

**Authors:** Nardi Tetaj, Andrea Segreti, Aurora Ferro, Virginia Ligorio, Alberto Spagnolo, Francesco Grigioni

**Affiliations:** 1 Cardiology Unit, Campus Bio-Medico Hospital University, Via Alvaro del Portillo 200, 00128 Roma, Italy; nardi.tetaj@unicampus.it (N.T.); aurora.ferro@unicampus.it (A.F.); virginia.ligorio@unicampus.it (V.L.); alberto.spagnolo@unicampus.it (A.S.); f.grigioni@policlinicocampus.it (F.G.); 2 Research Unit of Cardiovascular Science, Department of Medicine and Surgery, Campus Bio-Medico Hospital University, Via Alvaro del Portillo 21, 00128 Roma, Italy

**Keywords:** cardiomyopathy, sudden cardiac death, ventricular arrhythmias, genotype–phenotype, LMNA, FLNC, PLN, RBM20, desmoplakin, desmosomal genes, ICD, cardiac MRI, late gadolinium enhancement

## Abstract

Inherited cardiomyopathies represent a major cause of ventricular arrhythmias (VA) and sudden cardiac death (SCD), frequently occurring in the absence of advanced systolic dysfunction. Traditional strategies for the primary prevention of SCD have relied predominantly on left ventricular ejection fraction (LVEF), an approach that fails to capture the substantial biological and clinical heterogeneity of non-ischemic cardiomyopathies. Over the past decade, advances in cardiac genetics and cardiac magnetic resonance imaging have identified specific genotypes associated with a disproportionate arrhythmic risk, which often precedes overt ventricular remodeling. The 2023 European Society of Cardiology (ESC) Guidelines on cardiomyopathies formalize this paradigm shift by integrating etiology, myocardial substrate, and electrical phenotype into contemporary risk stratification. In this narrative review, we focus on cardiomyopathy-associated genotypes consistently linked to high arrhythmic risk—LMNA, truncating variants in FLNC, RBM20, PLN p.Arg14del, and desmosomal genes—and examine their molecular mechanisms, phenotypic trajectories, and arrhythmogenic profiles. We discuss how genotype-specific patterns of myocardial fibrosis, conduction disease, and VA inform implantable cardioverter-defibrillator (ICD) decision-making beyond LVEF-based thresholds. By synthesizing genetic, imaging, and clinical evidence in light of ESC 2023 recommendations, this review highlights the evolving role of genotype-informed strategies in the personalized prevention of SCD and underscores remaining gaps in evidence and risk prediction.

## 1. Introduction

Inherited cardiomyopathies are an important cause of heart failure, malignant ventricular arrhythmias (VA), and sudden cardiac death (SCD) across the age spectrum. Primary prevention of SCD in non-ischemic cardiomyopathies has long been based on global systolic dysfunction, typically using LVEF ≤ 35% after optimization of medical therapy as the principal criterion to consider ICD implantation [1]. This approach standardized practice, but it also implicitly treated non-ischemic cardiomyopathy as a relatively homogeneous entity, despite increasingly clear biological heterogeneity in arrhythmic substrate, scar distribution, and disease trajectories. Over the last decade, two convergent developments have challenged the adequacy of LVEF alone: the rapid maturation of cardiomyopathy genetics, revealing genotypes with disproportionate arrhythmic risk relative to global remodeling, and the expanding evidence base for CMR-based substrate characterization, particularly late gadolinium enhancement (LGE) as a correlate of VA and adverse outcomes in non-ischemic settings [2,3].

The 2023 ESC cardiomyopathy guidelines reflect this evolution by embedding etiology, including genotype, into risk assessment and by elevating myocardial tissue characterization, especially scar/fibrosis detected by CMR-LGE, as a central risk modifier. Within this framework, arrhythmic prevention becomes less about crossing a single functional threshold and more about identifying and tracking the convergence of cause (high-risk genotypes), substrate (scar/inflammation), and electrical expression (non-sustained ventricular tachycardia (NSVT), ectopy burden, syncope, and conduction disease) [4].

In this review, we synthesize current evidence on five major high-risk cardiomyopathy genotypes, LMNA, FLNC truncating variants, RBM20, PLN and desmosomal genes, with a focus on their arrhythmic phenotype, mechanisms of ventricular arrhythmogenesis and implications for clinical management in the ESC 2023 era. We integrate data from gene–disease validity curation and consensus guidelines to provide a practical, genotype-based framework for arrhythmic risk assessment, ICD decision-making in patients with inherited cardiomyopathies. The practical question addressed by this review is when genotype-positive patients should move from routine follow-up to intensified rhythm surveillance, repeat CMR, electrophysiology evaluation, and shared ICD discussion despite LVEF remaining above conventional thresholds.

## 2. Methods

**Figure 1:**
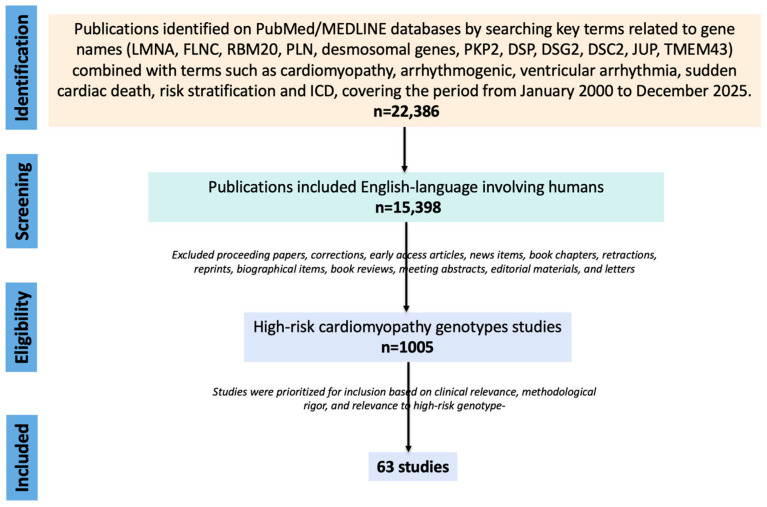
Literature search and study selection workflow for the narrative review.

A narrative literature search was conducted using PubMed/MEDLINE database to identify studies published from January 2000 up to December 2025. The review focused on genotypes with the most consistent evidence of arrhythmic risk disproportionate to LVEF, clinical relevance within the ESC 2023 framework, and practical applicability to surveillance or ICD decision-making. Search terms combined gene names (LMNA, FLNC, RBM20, PLN, desmosomal genes, PKP2, DSP, DSG2, DSC2, JUP, and TMEM43) with terms such as cardiomyopathy, arrhythmogenic, ventricular arrhythmia, sudden cardiac death, risk stratification and ICD. Genes not discussed in equivalent depth, including TTN truncating variants and selected non-desmosomal ACM-related genes, were not excluded because they lack relevance, but because available data are currently more phenotype-specific, less externally validated, or less consistently actionable for arrhythmic risk prediction across clinical settings. We excluded proceeding papers, corrections, early access articles, news items, book chapters, retractions, reprints, biographical items, book reviews, meeting abstracts, editorial materials, and letters. We included relevant English language studies involving humans, with a focus on cohorts and registries, risk-score derivation/validation studies, gene–disease validity papers, and ESC/consensus documents. Because this work was conceived as a narrative review, studies were not selected through formal meta-analytic procedures; instead, publications were prioritized according to clinical relevance, methodological rigor, and specific relevance to high-risk genotype, as shown in Figure 1.

## 3. ESC 2023 Framework for Genotype-Based Arrhythmic Risk Stratification

The 2023 ESC Guidelines for the management of cardiomyopathies represent a pragmatic turning point in arrhythmic risk assessment by explicitly embedding etiology, including genetic findings, into the pathway that leads from diagnosis to prevention of SCD. Rather than positioning LVEF as the principal gatekeeper for primary prevention ICD implantation, the guideline framework encourages an integrated appraisal of phenotype and disease stage, VA burden and conduction/atrial arrhythmias, myocardial tissue characterization (particularly scar/fibrosis), and genotype/family history [4].

This approach is conceptually aligned with the ESC 2022 VA/SCD Guidelines, which emphasize multiparametric risk evaluation and acknowledge that non-ischemic cardiomyopathy populations encompass heterogeneous SCD risk trajectories that are incompletely captured by LVEF alone [5].

A central innovation of the ESC 2023 is the systematic distinction between phenotype (e.g., DCM, ACM, HCM) and cause (genetic vs. non-genetic and specific gene/variant class when available), with clinical management, including arrhythmic prevention, being modulated by both axes. In practice, this means that genetic testing is no longer framed solely as a diagnostic label for family screening, but as a prognostic modifier that can reshape surveillance intensity and the timing of ICD discussions, particularly where arrhythmias can precede advanced structural remodeling [6].

This shift is especially relevant for inherited DCM, NDLVC, and left-dominant arrhythmogenic phenotypes, where gene-specific substrates can drive early electrical instability and fibrotic remodeling [7]. ESC 2023 discusses a subset of genes repeatedly associated with disproportionate VA/SCD risk relative to global systolic impairment, thereby functioning as risk enhancers. The most clinically actionable examples include LMNA, FLNC truncating variants, RBM20, PLN p.Arg14del, and desmosomal genes, particularly DSP as an archetypal left-dominant arrhythmogenic genotype [8,9,10]. Importantly, this does not imply that genotype alone is determinative; rather, the presence of a high-risk genotype should increase clinical vigilance, lower the threshold for additional investigations (CMR and extended rhythm monitoring), and support shared decision-making about ICD therapy when other adverse features coexist [11].

A second cornerstone of this guideline paradigm is the centrality of myocardial tissue characterization, most notably LGE as a surrogate marker of replacement fibrosis/scar and an established correlate of VA risk in multiple cardiomyopathy settings. This is particularly consequential for patients with preserved or mildly impaired LVEF, where LGE may reveal a high-risk substrate despite modest global dysfunction [12].

In parallel, ESC 2023 formally recognizes NDLVC as a distinct clinical entity, defined by non-ischemic LV scarring/lipomatous replacement without LV dilatation (with or without systolic dysfunction), or isolated global LV hypokinesia without scar and without dilatation. This classification provides a guideline “home” for patients, often gene carriers, such as FLNC, LMNA, or DSP, who manifest arrhythmias and/or LGE before fulfilling conventional dilated cardiomyopathy (DCM) criteria, thereby legitimizing earlier consideration of arrhythmic risk [13,14].

### 3.1. LMNA Cardiomyopathy—Prototypical “Electrical-First” High-Risk DCM/NDLVC Genotype

The LMNA gene, located in chromosome 1, encodes lamins A/C, nuclear-envelope proteins essential for nuclear structural integrity, chromatin organization, and gene regulation [15]. Pathogenic LMNA variants cause a spectrum of disorders (“laminopathies”) including muscular dystrophies, lipodystrophy, neuropathies and LMNA-related cardiomyopathy, often with overlapping phenotypes [16]. LMNA variants represent one of the highest-risk DCM genotypes, representing 5–10% of all DCM patients, second only to TTN-truncating variants, and characterized by early atrial and VAs, premature conduction disease, sustained VT/VF and/or appropriate ICD therapies often occurring before severe systolic dysfunction. Penetrance increases with age, many carriers are asymptomatic in early adulthood but develop conduction disease or arrhythmias in their 30s–40s, with LV dysfunction following thereafter [17,18]. LMNA is repeatedly associated with disproportionate VA/SCD risk relative to remodeling. This “electrical-first” trajectory explains why LVEF alone is an imperfect gatekeeper for arrhythmic prevention in LMNA carriers [19,20,21]. Non-missense variants generally confer a worse prognosis than some missense variants [22,23].

Mid-wall septal LGE on CMR has been repeatedly associated with laminopathy and is highlighted in the ESC 2023 framework as a tissue signature that may orient etiological suspicion [24]. Importantly, myocardial fibrosis in LMNA carriers often develops early in the disease course and may be detectable before significant left ventricular dilation or severe systolic impairment [16,25].

Structured predictors have been incorporated into validated model (LMNA risk-VTA; https://lmna-risk-vta.fr(accessed on 30 January 2026), including male sex, AV block, NSVT, non-missense variant type, and declining LVEF, to estimate a 5-year risk of life-threatening ventricular tachyarrhythmias. This prognostic model was derived from a multicenter cohort of 444 patients with LMNA mutations (C-index 0.77) with a 5-year estimated risk threshold ≥ 7% predicted 96.2% of malignant VAs, which significantly facilitated the choice of candidates for ICD, supporting individualized prevention strategies [26,27]. Accordingly, the 2022 ESC guidelines on the prevention of SCD state that a primary ICD should be considered (Class IIa) in DCM/HNDCM with a pathogenic LMNA variant, when the estimated 5-year malignant VA risk (MVA) is ≥10% and at least one cardiac abnormality is present (LVEF < 50% and/or atrioventricular block and/or NSVT) [1]. The ESC 2023 framework explicitly lists LMNA among high-risk genotypes and points clinicians toward risk estimation using an LMNA-specific risk score alongside CMR fibrosis as part of SCD prevention decision-making [4].

Risk modifiers. In LMNA cardiomyopathy, arrhythmic concern is higher in carriers of non-missense variants and in those with NSVT, conduction disease/AV block, progressive LV dysfunction, or septal mid-wall LGE. The combination of genotype, conduction abnormalities, VAs, and fibrosis is supported by cohort-derived evidence and underpins current LMNA-specific risk prediction models. In clinical practice, a shared ICD discussion should be strongly considered when these features cluster, even before severe systolic dysfunction develops.

### 3.2. Filamin-C Truncating Variants (FLNCtv)—“Scar-First” High-Risk NDLVC/DCM Genotype with Disproportionate VA/SCD Risk

Filamin-C truncating variants (FLNC) encodes filamin-C, an actin-binding protein highly expressed in striated muscle, localized to the Z-disk and intercalated disks, where it supports sarcomeric integrity, mechano-sensing/signaling, cytoskeleton–membrane coupling and myofibrillar repair [28,29]. Pathogenic cardiomyopathy-associated FLNC variants are predominantly truncating variants (nonsense, frameshift, and splice) and typically act through haploinsufficiency (loss of functional protein) rather than toxic aggregation (more characteristics of some skeletal myopathy variants), plausibly favoring fibrotic remodeling. These FLNC truncating variants (FLNCtv) are consistently linked to an arrhythmogenic cardiomyopathy (ACM) and DCM phenotype [30,31]. Clinically, FLNCtv carriers typically show non-ischemic LV systolic dysfunction with only modest LV dilatation [32], extensive mid-wall, subepicardial fibrosis or circumferential “ring-like” LGE on CMR [33,34], and a high burden of ventricular ectopy/NSVT, with sustained VT/VF or SCD occasionally representing the first manifestation [35]. Onset is often in mid-adulthood, though both earlier and later presentations are reported. Inheritance is usually autosomal dominant with age-dependent penetrance, and the overall profile supports classifying FLNCtv as a high-risk arrhythmogenic DCM genotype [36]. A recently developed risk model that integrates age, male sex, NSVT, syncope, and LVEF has shown good performance in predicting life-threatening VAs in a large international cohort of FLNC truncating-variant carriers (https://flnctv.shinyapps.io/RiskCalculator/, accessed on 30 January 2026). The study found that these individuals carry a substantial arrhythmic risk, with 19% out of these individuals who experienced SCD or MVAs over a median 34-month follow-up, corresponding to about four events per 100 person-years. The risk was especially higher in probands and phenotype-positive carriers, while phenotype-negative carriers had a much lower event rate. While external validation remains pending, it can still offer helpful guidance for refining an individual patient’s risk profile within a comprehensive multiparametric evaluation [37].

Risk modifiers. In FLNC-associated cardiomyopathy, risk is most consistently linked to truncating variants, often in the setting of a “scar-first” phenotype. Key markers of concern include NSVT, frequent ventricular ectopy, syncope, progressive LV dysfunction, and non-ischemic LGE, particularly when extensive or distributed in subepicardial or ring-like patterns. Although a genotype-specific risk model has recently been proposed, external validation remains limited; therefore, ICD discussion should be framed within a multiparametric assessment rather than a single-threshold approach.

### 3.3. RBM20 Cardiomyopathy—Aggressive, “Arrhythmia-and-HF–Prone” DCM Genotype

RNA Binding Motif Protein 20 (RBM20) encodes an RNA-binding splicing regulator that is pivotal for correct processing of key cardiomyocyte transcripts, most notably titin (TTN)**,** but also several ion-channel and calcium-handling genes, so pathogenic variants can derail splicing programs, shift titin isoform balance, perturb calcium handling, and drive progressive ventricular remodeling toward an arrhythmogenic DCM/ALVC phenotype [38,39]. Most cardiomyopathy-associated RBM20 variants are missense “hot-spot” changes, e.g., RS-rich domain and around specific exons (exon 9 and 11), with stronger evidence for pathogenicity and risk [40]. These regions are essential for nuclear localization and RNA binding. Outside these hot spots, the likelihood that an RBM20 variant is truly pathogenic is lower [41]. Truncating RBM20 variants in TTN (TTNtv) represent the most prevalent monogenic cause of DCM (up to 25%), but it confers milder disease severity alone than RBM20 pathogenic/likely pathogenic (P/LP) variants [42,43]. In clinical practice, RBM20 disease is typically autosomal dominant with variable (often high, age-dependent) penetrance and, in many cohorts, a marked male predominance, with early and frequent VAs (NSVT, sustained VT/VF, and appropriate ICD therapies) alongside rapid progression to advanced heart failure [44,45]. Age at onset often falls in early to mid-adulthood, although adolescent presentations have been described [46]. In several series, RBM20 cardiomyopathy shows a “double hit”: early malignant arrhythmias plus a high rate of transition to end-stage HF, making it one of the most ominous genotypes in the non-syndromic DCM spectrum [47]. In contrast to LMNA and FLNC, RBM20-associated cardiomyopathy does not exhibit a single pathognomonic LGE pattern [4,48]. CMR findings are more heterogeneous, ranging from patchy or diffuse mid-wall fibrosis to minimal or absent LGE in early stages [45,46,47,49]. RBM20 is incorporated into the ESC 2023 high-risk genotype concept, acknowledging genotype-mediated risk beyond an LVEF-only paradigm. Combined early CMR-based scar assessment and structured rhythm surveillance refine risk. Unlike other high-risk genes, there are no validated RBM20-specific ICD risk calculators, reason why developing and validating such models will be crucial for standardizing decisions across centers [49].

Risk modifiers. In RBM20 cardiomyopathy, higher concern is warranted for pathogenic variants affecting recognized hotspot regions, especially when accompanied by early NSVT or sustained VTs, family history of sudden cardiac death, and evidence of non-ischemic fibrosis on CMR. Because no validated RBM20-specific ICD calculator is currently available, escalation to intensified surveillance and ICD discussion is based mainly on the convergence of genotype, electrical instability, and evolving myocardial substrate rather than on a formal risk score.

### 3.4. PLN Cardiomyopathy—Early Fibrosis and High VA/SCD Risk

PLN encodes phospholamban, a small transmembrane protein in the sarcoplasmic reticulum that regulates SERCA2a and thus sarcoplasmic Ca^2+^ uptake during diastole. In its dephosphorylated state, phospholamban inhibits SERCA2a; phosphorylation relieves this inhibition and enhances Ca^2+^ reuptake, providing a plausible substrate for electrical instability and fibrotic remodeling [50]. Four different (likely) pathogenic variants in this gene have been listed (p.Arg9Cys, p.Arg9His, p.Leu39*, and p.Arg14del) [51,52]. Of these, the best-characterized cardiomyopathy-associated variant is the founder deletion p.Arg14del, first described in families with lethal hereditary cardiomyopathy and subsequently confirmed as a definitive cause of inherited cardiomyopathy across multiple cohorts, with an autosomal-dominant pattern and a notable founder effect in certain populations (particularly in the Netherlands and neighboring regions) [53,54]. From a clinical perspective, PLN p.Arg14del is now regarded as a prototypical high-risk genotype producing a mixed dilated/arrhythmogenic LV phenotype (often framed within the DCM–NDLVC/ALVC spectrum), with a broad range of expression from apparently asymptomatic carriers with subtle ECG abnormalities to patients with recurrent VT/VF or progression to end-stage DCM [55,56]. Characteristic ECG clues include very low QRS voltages and T-wave inversion, while CMR commonly reveals non-ischemic fibrosis that helps contextualize arrhythmic risk [57,58]. Age at onset is usually in young to middle adulthood, but SCD can occur earlier, and penetrance increases with age [59,60]. The PLN p.Arg14del mutation is associated with a distinctive CMR phenotype, frequently characterized by subepicardial or ring-like LGE involving the left ventricle [61,62]. In a cohort of 679 PLN p.Arg14del carriers followed for a median of 4.3 years, 72 (10.6%) experienced malignant VAs. A mutation-specific risk score was derived using four routine markers, LVEF, 24 h PVC burden, extent of T-wave inversion, and low-voltage ECG, with strong performance in the derivation cohort (optimism-corrected C-index 0.83) and good risk separation in landmark analyses. Online calculators are available at https://plnriskcalculator.shinyapps.io and https://www.acm-risk.com/pln_risk.html (both URLs accessed on 30 January 2026), although externally unvalidated, and can support ESC-era multiparametric risk profiling and shared ICD decision-making [63].

Risk modifiers. In PLN p.Arg14del cardiomyopathy, arrhythmic risk is heightened by ventricular ectopy or NSVT, low-voltage ECG or repolarization abnormalities, progressive LV dysfunction, and non-ischemic fibrosis on CMR, often with subepicardial or ring-like LGE. Available cohort-based models support a genotype-informed approach, although interpretation should remain phenotype-aware. When electrical instability and fibrotic substrate coexist, shared ICD discussion may be appropriate even outside conventional LVEF-based thresholds.

### 3.5. Desmosomal Genes and Arrhythmogenic Cardiomyopathy Spectrum

Desmosomes are intercellular junctions that provide mechanical coupling between cardiomyocytes, particularly in the right ventricle and subepicardial regions subject to high mechanical stress [64]. Pathogenic variants in desmosomal genes, most prominently plakophilin-2 (PKP2), desmoplakin (DSP), desmoglein-2 (DSG2), desmocollin-2 (DSC2), and plakoglobin (JUP), disrupt cell–cell adhesion, gap-junction organization and intracellular signaling, leading to myocyte detachment, apoptosis and fibro–fatty replacement, the histological hallmark of arrhythmogenic right ventricular cardiomyopathy (ARVC) and broader arrhythmogenic cardiomyopathy (ACM) [65,66,67]. Desmosomal disease is now widely recognized as a biventricular or left-dominant spectrum, in which VA/SCD risk often tracks more closely with scar/inflammatory activity and arrhythmic history than with LVEF alone [7,68].

Desmosomal cardiomyopathies often show a characteristic ECG pattern with T-wave inversion in V1–V3 (often extending to lateral leads in left-dominant disease), epsilon waves or prolonged terminal activation in right precordial leads, and frequent low QRS voltages or QRS fragmentation [69,70]. VAs are common and typically display a left bundle branch block morphology, reflecting right ventricular origin and an early arrhythmogenic substrate [71].

In cardiomyopathies related to desmosomal genes, CMR findings vary according to whether the phenotype is predominantly ARVC or left-dominant/biventricular disease, as commonly observed with desmoplakin (DSP) variants [72,73]. While PKP2 remains prototypical for classic ARVC, DSP has emerged as an archetype for left-dominant/biventricular ACM, characterized by early LV involvement, inferolateral subepicardial, “ring-like” or mid-wall LGE, and “myocarditis-like” episodes that may evolve into fibrosis and electrical instability [74,75,76,77]. This inflammation-to-scar trajectory explains why many desmosomal carriers are present in the NDLVC/left-dominant ACM space, with significant LGE and/or VAs before dilatation or severe dysfunction [78,79]. The classical phenotype predominantly involves the RV (hence ARVC). But over time, thanks to the widespread use of cardiac magnetic resonance (CMR), genetic testing, and post-mortem investigations, it has been recognized that the LV is also frequently involved. These variants are characterized by biventricular or even LV-predominant involvement [80]. Despite major progress in recognizing the broader ACM spectrum, the classification and risk stratification of left-dominant and biventricular phenotypes remain an evolving area, and the relationship between traditional ARVC criteria and newer phenotype-based frameworks continues to be debated [11]. In fact, by doing so, while ARVC with a predominant right ventricular phenotype and some left ventricular involvement remains labeled as ‘ARVC’, biventricular and left-dominant ventricular phenotypes fall under the NDLVC definition [4,81].

The 2023 ESC cardiomyopathy guidelines acknowledge this phenotypic overlap, using the broader term ACM to encompass RV, biventricular and LV-dominant forms linked by a common arrhythmogenic substrate. Pragmatic “electrical + imaging + history” risk markers include NSVT/high PVC burden, unexplained syncope, prior sustained VT/VF, LV involvement, and scar burden/pattern on CMR [82]. A distinctive feature of desmosomal ACM is an evidence that high-intensity endurance exercise increases disease penetrance and arrhythmic events, particularly in classic ARVC/PKP2 contexts. Exercise counseling, therefore, becomes a modifiable risk lever [83]. In ESC 2023-era logic, desmosomal genes, especially those linked to left-dominant phenotypes (notably DSP), fit naturally into the high-risk genotype concept, strengthening ICD discussions when genotype coexists with syncope, NSVT, progressive scar, or ventricular dysfunction [84]. DSP risk score (www.DSP-risk.com, accessed on 30 January 2026), including female sex, NSVT, PVC burden, LVEF < 50% and moderate or severe right ventricular dysfunction. The model derives from a recent developed and externally validated DSP-specific risk score for first sustained ventricular arrhythmias in 471 carriers of pathogenic/likely pathogenic DSP variants, with good accuracy (C-index 0.78). Over a median 4-year follow-up, 71 patients had an arrhythmic event (annual rate 2.6%). Clinically, the model may support individualized discussion but require careful interpretation in evolving phenotypes and local calibration [85].

In addition, the ARVC Risk Calculator represents a major advance in individualized arrhythmic risk stratification for patients with ARVC. Developed from large, multicentre cohorts, it estimates the 5-year risk of sustained VAs using readily available clinical variables, including age, sex, history of syncope, number of T-wave inversions, premature ventricular complex burden, non-sustained ventricular tachycardia, and right ventricular function, rather than relying on dichotomous criteria alone [86].

Nevertheless, important limitations remain: the score was developed primarily in patients fulfilling definite ARVC criteria, largely with classical right-dominant disease, limiting its applicability in left-dominant or biventricular phenotypes, early genotype-positive individuals, and non-desmosomal ACM [87,88].

Importantly, the calculators outperform traditional guideline-based algorithms in predicting arrhythmic events and support a personalized approach to ICD decision-making, particularly in genotype-positive or early-stage disease, aligning with the contemporary shift toward continuous risk models in inherited cardiomyopathies.

Importantly, several gray zones persist in ACM, particularly with respect to non-desmosomal disease genes. A paradigmatic example is TMEM43, especially the p.S358L mutation, which encodes a transmembrane protein localized to the plasma membrane, nuclear envelope, and endoplasmic reticulum, and has been consistently associated with very high arrhythmic events rates [89,90].

Risk modifiers. In desmosomal cardiomyopathies, risk interpretation should remain phenotype-specific, particularly when extending beyond classic right-dominant ARVC to left-dominant or biventricular forms. Higher concern is associated with NSVT, high PVC burden, unexplained syncope, ventricular dysfunction, and non-ischemic LGE, especially inferolateral subepicardial or ring-like patterns in DSP-related disease. Existing calculators derived from ARVC cohorts can support decision-making, but their applicability to broader ACM phenotypes is incomplete; therefore, ICD discussion should integrate genotype, phenotype, electrical expression, and scar burden rather than relying on ARVC-derived thresholds alone.

**Figure 2:**
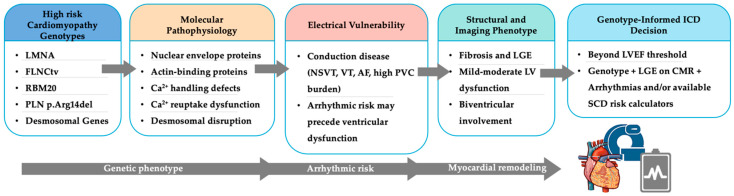
Conceptual framework for genotype-driven arrhythmic risk stratification in inherited cardiomyopathies. Abbreviations: NSVT, non-sustained ventricular tachycardia; VT, ventricular tachycardia; AF, atrial fibrillation; PVC, premature ventricular contractions; LGE, late gadolinium enhancement; CMR, cardiac magnetic resonance; LV, left ventricular; SCD, sudden cardiac death.

Taken together, these high-risk genotypes converge on a common clinical pathway: gene-specific molecular abnormalities promote fibrosis, electrical instability, and ventricular remodeling, generating an arrhythmogenic substrate that may be detectable by CMR and rhythm monitoring before advanced LV dysfunction. This concept is summarized in Figure 2. These upstream mechanisms promote myocardial remodeling and fibrosis, which can be detected by CMR imaging as non-ischemic LGE, often in the setting of mild or moderate ventricular dysfunction. The resulting arrhythmogenic substrate confers an increased susceptibility to malignant VAs and SCD, frequently disproportionate to global systolic impairment.

**Figure 3:**
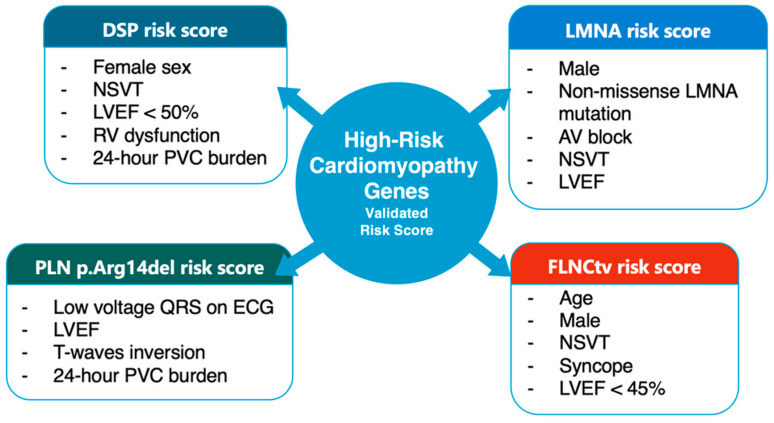
Overview of genotype-specific arrhythmic risk scores in high-risk cardiomyopathy genes, highlighting the distinct clinical and electrocardiographic predictors used for risk stratification in DSP, LMNA, FLNC truncating variants, and PLN p.Arg14del cardiomyopathies. Abbreviations: NSVT, non-sustained ventricular tachycardia; AV block, atrio-ventricular block; LVEF, left ventricular ejection fraction; PVC, premature ventricular contractions; RV, right ventricle.

In the 2023 ESC cardiomyopathy guidelines, integration of genotype, imaging phenotype, and electrical markers supports individualized implantable cardioverter-defibrillator (ICD) decision-making beyond left ventricular ejection fraction (LVEF)–based thresholds. For each genotype, distinct clinical, electrocardiographic, and imaging variables contribute to risk prediction models, reflecting divergent arrhythmogenic mechanisms. Figure 3 summarizes currently available, genotype-specific arrhythmic risk scores for selected high-risk cardiomyopathy genes. These scores integrate markers of electrical instability, ventricular dysfunction, and conduction disease to refine arrhythmic risk stratification and support individualized decision-making for primary prevention of SCD beyond LVEF-based criteria.

## 4. Practical Workflow for Genotype- and Scar-Informed VA/SCD Prevention (ESC 2023 Era)

Across the gene-specific sections reviewed above, a consistent message emerges in non-ischemic cardiomyopathy and arrhythmogenic phenotypes; VA and SCD risk are frequently not captured adequately by LVEF alone. The ESC 2023 cardiomyopathy framework reflects this reality by placing prevention decisions within an integrated assessment that combines phenotype, etiology (including genotype), myocardial substrate (scar and/or inflammation), and arrhythmic expression. Practically, this means that clinicians are no longer asked to identify only “who has a low EF,” but rather to recognize who has an arrhythmogenic substrate, who is already expressing electrical instability, and who is likely to transition from “risk” to “event” unless surveillance and/or prophylactic therapy is intensified.

Across the gene-specific sections reviewed above, a consistent message emerges: in inherited non-ischemic cardiomyopathies, VA/SCD risk is often not adequately captured by LVEF alone. In the ESC 2023 framework, prevention decisions should therefore integrate entry phenotype, genotype, myocardial substrate, and electrical expression, allowing clinicians to identify patients with evolving arrhythmogenic risk before advanced systolic dysfunction becomes evident.

**Figure 4:**
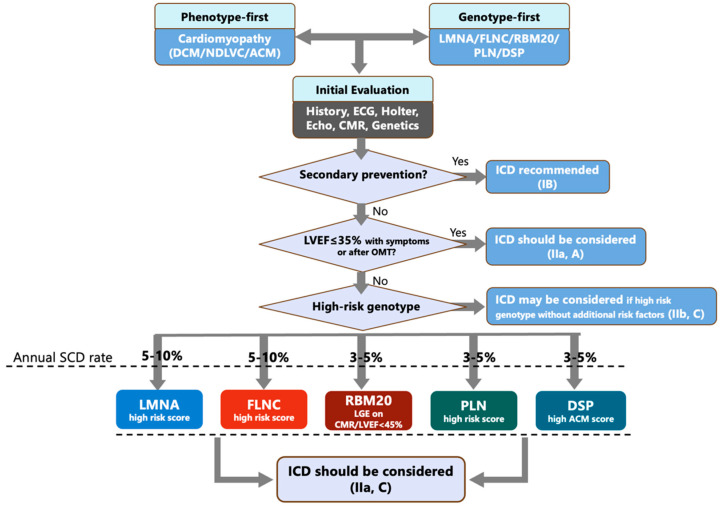
Conceptual framework for high-risk genotype-driven algorithm for sudden cardiac death prevention in inherited cardiomyopathies. Abbreviations: DCM, dilated cardiomyopathy; NDLVC, non-dilated left ventricular cardiomyopathy; CMR, cardiac magnetic resonance; SCD, sudden cardiac death; ECG, electrocardiogram; LVEF, left ventricular ejection fraction; and ICD, implantable cardioverter defibrillator.

The 2022 ESC VA/SCD guideline recommends CMR with LGE to refine diagnosis and arrhythmic risk in the genetic work-up of DCM/HNDCM. Crucially, it endorses a multiparametric primary-prevention approach in which an ICD should be considered not only for persistent LVEF ≤ 35% despite optimal therapy, but also in patients with LVEF < 50% plus ≥ 2 risk factors, including syncope, LGE on CMR, inducible SMVT at EPS, and pathogenic variants in high-risk genes. Figure 4 depicts a genotype-informed ICD decision pathway in inherited cardiomyopathies, integrating phenotype-first and genotype-first approaches. The clustering of high-risk genotype-specific features may support ICD consideration according to an ESC-aligned framework (Class IIa, Level of Evidence C). Beyond secondary prevention and reduced LVEF threshold, the presence of high-risk genotypes identifies patients with substantial arrhythmic risk, supporting ICD consideration.

The concept of disease staging has long been central to heart failure management and hypertrophic cardiomyopathy, where temporal progression from genotype carriage to overt structural disease is well recognized. A pragmatic first step to define the patient’s entry phenotype and disease stage may enhance the meaning of risk markers, and the downstream pathway, which differs across DCM, NDLVC, and ACM. In DCM, remodeling and systolic impairment are prominent, but scar burden may vary widely. In NDLVC, non-ischemic scar and/or global hypokinesia can exist without LV dilatation, often with only mild functional impairment; this is particularly relevant for FLNCtv, LMNA, DSP and PLN carriers. A practical NDLVC entry profile includes one or more of the following: non-ischemic LV LGE in the absence of significant coronary disease, mildly reduced LV systolic function without overt dilatation, frequent PVCs or NSVT disproportionate to structural severity, unexplained syncope, or genotype-positive status with evolving electrical abnormalities. In such patients, surveillance intensity should be escalated according to risk clustering: periodic Holter or extended ambulatory monitoring, earlier repeat CMR when a scar is present or symptoms/electrical markers evolve, and electrophysiology referral when NSVT, rising ectopy burden, syncope, conduction disease, or progressive LGE coexist. In the ACM spectrum, the dominant driver of malignant arrhythmias may be scar and inflammatory activity rather than global LV systolic function.

Alongside phenotype, recording a short “stage statement” (stable vs progressive; early expression vs advanced HF; and isolated electrical disease vs combined electrical and HF progression) may help frame-competing risks and ICD net benefit, as shown in an example in Table 1. Future ESC updates and risk models are likely to move toward this paradigm, particularly as longitudinal cohort data and genotype-specific risk scores mature [14,91]. In this context, staging may serve as a unifying framework linking molecular genetics to clinical decision-making across the disease lifespan. In high-risk genes atrioventricular conduction disease, atrial arrhythmias, and ventricular ectopy are recognized as early disease manifestations, often preceding left ventricular dilation or systolic impairment [92]. These features are increasingly interpreted as markers of disease progression rather than isolated findings.

Once phenotype is established, ESC 2023 encourages clinicians to ask, “why this phenotype?” and to document cause explicitly rather than defaulting to phenotype-only risk stratification. Beyond the binary presence of a pathogenic/likely pathogenic variant, useful documentation includes the gene involved, variant class when relevant (e.g., LMNA non-missense variants, and FLNCtv), and plausible domain/hotspot implications (e.g., RBM20 regions linked to aggressive disease). Family history should be captured in a structured way, because clustering of premature SCD, sustained VT/VF, ICD implantation, unexplained syncope, or early transplant/advanced HF often shifts thresholds for monitoring and prophylactic discussions [92]. This “cause” step should also include modifiable or amplifying factors that interact with genotype, such as high-intensity endurance exercise in desmosomal disease and myocarditis-like inflammatory episodes in DSP-associated phenotypes. Within ESC 2023 logic, genotype is best treated as a risk enhancer that lowers the threshold for deeper evaluation (CMR and extended monitoring), rather than as a stand-alone ICD indication.

With phenotype and cause clarified, the next task is to quantify arrhythmic expression, the patient’s “electrical load.” Baseline ECG and ambulatory monitoring are central. Pragmatic red flags include recurrent or fast NSVT, high PVC burden or complex ectopy, and unexplained syncope (particularly recent, recurrent, or exertional). Atrial fibrillation/flutter may be a relevant risk signal in settings where it clusters with malignant ventricular disease (e.g., LMNA and RBM20) [94]. Where initial monitoring is non-diagnostic, but suspicion remains high (such as genotype-positive patients with symptoms, NDLVC patients with substantial scar but “quiet” routine monitoring, or clustered risk markers without documentation), there should be a low threshold to escalate to prolonged external monitoring or, in selected cases, implantable loop recording [95]. A key point is that electrical assessment is rarely “one-and-done”; repeating monitoring over time may be more informative than a single snapshot.

The ESC 2023 framework places major emphasis on defining the myocardial substrate, particularly through CMR-LGE, because in non-ischemic disease scar burden and pattern often track VA risk more closely than LVEF does. To make CMR findings clinically actionable for arrhythmic risk discussion, LGE reporting should include at minimum: presence or absence, distribution/pattern (e.g., septal mid-wall, inferolateral subepicardial, circumferential/ring-like), approximate extent (by segments involved or percentage of LV mass when available), and interval progression on serial studies [96]. These elements are particularly important in genotype-positive patients with preserved or mildly reduced LVEF, in whom scar pattern may materially influence surveillance and ICD discussions. At the same time, LGE interpretation remains partly center-dependent, with variability related to sequence parameters, field strength, vendor platforms, thresholding methods, and reader expertise. When clinical features suggest active inflammation, particularly myocarditis-like DSP presentations, CMR evaluation for edema/inflammatory activity can help link episodes of injury to subsequent scar evolution and risk progression [96]. A practical heuristic aligned with several high-risk genotypes is to regard “mild LV dysfunction with substantial LGE” as a high-concern configuration, warranting intensified rhythm surveillance and earlier prophylactic discussions rather than reassurance based on LVEF above traditional thresholds.

Once phenotype, cause, electrical expression, and substrate are assembled, risk should be integrated explicitly. Where validated gene-specific tools exist (notably LMNA and PLN p.Arg14del), they can support transparent weighting of variables. Where tools are unavailable, unvalidated, or poorly calibrated to the patient’s phenotype, a structured “risk cluster” approach is a defensible alternative: convergence of (i) a genotype signal (high-risk gene/variant class), (ii) an electrical signal (NSVT, high ectopy, syncope, and sustained VA), (iii) a substrate signal (LGE presence/extent/pattern, progressive scar, and inflammation-to-scar phenotype), and (iv) a stage/trajectory signal (declining LVEF, ventricular enlargement, and worsening HF markers) [97]. Documenting integration in a single sentence (e.g., “high-risk genotype with LGE and NSVT implies high arrhythmic concern despite LVEF above the classic threshold”) improves consistency across multidisciplinary teams and strengthens shared decision-making. Table 2 synthesizes genotype-specific echocardiographic, electrocardiographic, and CMR features in high-risk cardiomyopathies, illustrating how distinct structural and electrical substrates underpin arrhythmic risk beyond LVEF.

The key operational step is translating risk into an action plan, best framed as “management intensity” rather than a binary ICD decision anchored solely to LVEF. Lower-intensity pathways may be appropriate when scar is absent/minimal, NSVT/high ectopy is absent, syncope is absent, and function is stable. Intermediate “monitor closely” pathways are typical when any of the following are present: high-risk genotype, meaningful LGE, rising ectopy, early conduction disease, or modest functional decline, prompting scheduled ambulatory monitoring, planned repeat CMR when a scar is dominant, early EP input as electrical markers evolve, and exercise counseling where relevant (especially desmosomal disease). High-intensity pathways are appropriate when multiple high-risk markers cluster—high-risk genotype plus LGE and/or NSVT/syncope, progressive scar or LV involvement, sustained VA, rapid functional deterioration, or strong family history—prompting prompt EP review and early shared decision-making about ICD timing consistent with ESC 2023 logic. Throughout, competing risks must remain visible in advanced HF and ICD candidacy, and timing should integrate HF trajectory and patient goals [97,99].

Finally, prevention is inherently longitudinal. Risk stratification should be conceptualized as a loop: repeat rhythm surveillance in high-risk genotypes and scar-positive phenotypes, repeat CMR when scar/inflammation-to-scar evolution is central, and cascade evaluation with staged phenotyping of relatives. A practical habit is to close each clinical encounter with a documented “reassessment trigger” (new NSVT, scar progression, syncope, and functional decline) that would prompt escalation or an ICD discussion, keeping proactive rather than reactive care [100].

## 5. Limitations and Pitfalls in Genotype- and Scar-Based VA/SCD Prevention

The 2023 cardiomyopathy guidelines issued by the European Society of Cardiology represent a major conceptual shift toward genotype- and imaging-informed prevention of VAs and SCD. However, several important limitations and potential pitfalls remain, which must be acknowledged to avoid overinterpretation and inappropriate clinical extrapolation.

A central limitation of genotype-based risk stratification is the marked heterogeneity in phenotypic expression. Much of the risk signal for high-risk genotypes derives from observational cohorts and registries, often enriched for severe phenotypes. This heterogeneity reflects, in part, the fact that penetrance is often incomplete, age-dependent, and strongly modified by context. Even within the same gene, arrhythmic risk may vary according to variant class or hotspot, sex, family history, myocardial scar burden, inflammatory activity, and external modifiers such as endurance exercise. Thus, the presence of a pathogenic or likely pathogenic variant should not be interpreted in a deterministic manner. Rather than functioning as a stand-alone predictor, genotype is best understood as a risk enhancer whose clinical significance depends on its interaction with phenotype, substrate, and electrical expression over time. This is particularly important in referral-center cohorts, which may enrich for more severe presentations and therefore overestimate penetrance or event rates when findings are generalized to broader genotype-positive populations. VAs may occur early, late, or not at all, despite the presence of a pathogenic variant. This variability limits the predictive precision of genotype alone and underscores the risk of deterministic interpretations of genetic findings. A single, baseline risk assessment may therefore be insufficient, yet standardized protocols for longitudinal re-stratification remain underdeveloped [101,102].

Gene-specific models represent a major advance, but their validity depends on calibration to the treated population. Differences in referral patterns, therapy era, imaging protocols, and endpoints can lead to miscalibration when models are applied outside derivation/validation contexts. This is particularly relevant when ARVC-derived tools are extrapolated to left-dominant DSP phenotypes or when emerging calculators are used before external validation is mature [95,103].

LGE on CMR has emerged as a cornerstone of arrhythmic risk assessment. Nevertheless, several pitfalls persist. Pattern recognition is clinically meaningful yet inconsistently reported, and dynamic biology matters: scar can progress, and inflammation-prone phenotypes introduce time-dependence rarely captured by a single scan. In addition, LGE presence and extent is influenced by technical factors, acquisition protocols, and post-processing methods, limiting inter-center reproducibility. On the other hand, the absence of LGE does not exclude arrhythmic risk, particularly in genotypes characterized by early electrical instability preceding overt fibrosis. Conversely, extensive LGE does not uniformly translate into malignant arrhythmias, especially in older or clinically stable individuals [62]. A pragmatic clinical goal might be reproducible reporting (presence, pattern, approximate extent, and change over time).

Lowering the threshold for ICD discussions is rational in high-risk genotypes, but harms (inappropriate shocks, lead failure, infection, and psychological burden) and long-term complications, especially in younger patients, must be weighed explicitly. Current recommendations are largely informed by observational cohorts and retrospective analyses. However, randomized controlled trials evaluating ICD implantation strategies specifically tailored to genotype and scar burden remain lacking [97,99]. As a result, guideline recommendations necessarily rely on expert consensus and indirect evidence, with an inherent risk of both over- and under-protection in selected patient subsets.

### Future Direction

Future prevention strategies are likely to move beyond binary thresholds toward continuous, dynamic risk models integrating genotype, scar characteristics, electrical markers, and clinical evolution. Scientific statements from the ESC Heart Failure Association emphasize the need for next generation arrhythmogenic risk scores capable of capturing disease progression over time rather than relying on static parameters [5].

Rather than a unified cardiomyopathy risk framework, future approaches may adopt gene- or pathway-specific algorithms. For example, the arrhythmic substrate in LMNA cardiomyopathy differs fundamentally from that observed in desmosomal disease or FLNC-related cardiomyopathy [16]. Tailored models may improve discrimination between patients who derive clear survival benefit from early ICD implantation and those in whom conservative monitoring is appropriate. Multidisciplinary cardiomyopathy clinics are ideal for genotype-aware care, but pathways must be scalable. Although not yet formalized in ESC guidelines, a genotype-driven staging system may represent a logical and necessary evolution of cardiomyopathy management. Such a model would integrate genetic substrate, electrical instability, myocardial fibrosis/remodeling, and heart failure progression. Future ESC updates and risk models are likely to move toward this paradigm, particularly as longitudinal cohort data and genotype-specific risk scores mature. In this context, staging may serve as a unifying framework linking molecular genetics to clinical decision-making across the disease lifespan.

Emerging CMR techniques, including quantitative tissue characterization, diffuse fibrosis mapping, and machine-learning-assisted scar analysis, may refine the prognostic value of imaging. Integration with digital phenotyping, such as continuous rhythm monitoring and wearable-derived metrics, could further enhance early detection of electrical instability [24,62].

The next phase of precision prevention may incorporate transcriptomic, proteomic, or functional assay data to complement DNA-based testing. Functional characterization of variants, particularly in genes with pleiotropic effects, may help distinguish truly malignant genotypes from variants with limited arrhythmic relevance [74,104].

Furthermore, expanding indications for genotype- and scar-driven ICD implantation raise important ethical and health-system questions. These include the psychological burden of labeling young or asymptomatic carriers as “high-risk,” equitable access to advanced imaging and genetic testing, and long-term device-related complications in populations with uncertain absolute risk.

Prospective contemporary cohorts with standardized protocols, external validation/recalibration of risk models (including left-dominant ACM phenotypes), improved substrate metrics (integrating LGE quantification with mapping and markers of inflammation), and integrated prediction approaches that combine genotype with longitudinal rhythm and substrate evolution are priorities. Patient-centered outcomes are also needed to refine shared decision-making about device therapy.

## 6. Conclusions

The ESC 2023 era reframes VA/SCD prevention in inherited cardiomyopathies from an LVEF-centered strategy to a multiparametric and longitudinal model integrating phenotype, genotype, myocardial substrate, and electrical expression. In high-risk genotypes such as LMNA, FLNC truncating variants, RBM20, PLN p.Arg14del, and desmosomal genes, clinically meaningful arrhythmic risk may emerge before severe systolic dysfunction and is often better captured by the convergence of genotype, scar, and electrical instability than by LVEF alone.

At the same time, penetrance remains variable and genotype should be interpreted as a probabilistic rather than deterministic signal, requiring longitudinal reassessment within a multiparametric framework. A practical, clinic-ready approach, therefore, requires consistent documentation of entry phenotype (including NDLVC when appropriate), structured capture of genotype and family history, repeated rhythm surveillance, careful CMR substrate characterization, and transparent integration using validated gene-specific tools where available or structured “risk cluster” logic where not. A key remaining challenge is to translate strong directional evidence into individualized estimates of absolute risk and net ICD benefit through better standardization, calibration, and prospective validation, while keeping shared decision-making central in a landscape where uncertainty remains substantial, but actionable signals are increasingly identifiable.

## Figures and Tables

**Table 1:** Gene-specific staging trajectories and arrhythmic risk in high-risk cardiomyopathy genotypes.

Gene	Early/Pre-Phenotypic Stage	Electrical-Predominant Stage	Structural Disease Stage	Advanced Disease Stage
*LMNA*	Asymptomatic carriers; normal imaging	Early AVB, atrial arrhythmias, NSVT often with preserved LVEF [23]	Mild LV dilation, myocardial fibrosis [20]	Progressive DCM, pacing dependency, sustained VT/VF, HF progression [16]
*FLNC (truncating variants)*	Usually silent until adulthood; rare childhood phenotype [28,30]	Frequent PVCs or NSVT may precede dilation; ECG abnormalities (low voltages, inferolateral T-wave inversion) [29]	LV dilation and systolic dysfunction with extensive inferolateral fibrosis [32]	High burden of malignant VAs, SCD risk often disproportionate to LVEF [31,35]
*RBM20*	Genotype-positive carriers may show early remodeling tendency [38,40]	VAs may occur early, sometimes before overt dilation [39,41,46]	Rapidly progressive DCM with fibrosis and high arrhythmic burden [45,47]	Early HF progression, transplant or LVAD requirement; recurrent VT/VF [44,49]
*PLN (p.Arg14del)*	Long preclinical phase common; subtle ECG changes [50,51]	Low QRS voltages, atrial arrhythmias, NSVT often precede LV dysfunction [52,57]	Mixed DCM/ACM phenotype with fibrosis and VAs [55,93]	Progressive HF with high arrhythmic mortality [58,60]
*Desmosomal genes*	Often asymptomatic carriers; variable penetrance [66,73]	Ventricular ectopy, T-wave inversion, epsilon waves (selected cases) [70,71,80]	Arrhythmogenic phenotype with RV and/or LV involvement; fibrosis [65,75,81]	Advanced biventricular disease, sustained VT/VF, HF progression [78,83]

Abbreviations: AVB, atrio-ventricular block; NSVT, non-sustained ventricular tachycardia; LV, left ventricle; VT, ventricular tachycardia; VF, ventricular fibrillation; HF, heart failure; DCM, dilated cardiomyopathy; and ACM, arrhythmogenic cardiomyopathy.

**Table 2:** Gene-specific echocardiographic, electrocardiographic, and CMR features in high-risk cardiomyopathy genotypes.

Gene	Echocardiographic Features	Electrocardiographic Features	Cardiac Magnetic Resonance Features	Arrhythmic Risk Implications (ICD Decision-Making)
*LMNA*	Mild–moderate LV dilation; early systolic impairment; clinical severity may be disproportionate to LVEF [16,20,25]	AV conduction disease (PR prolongation, AV block); atrial arrhythmias; NSVT; pacing dependency frequent [19,21,23]	Mid-wall/septal LGE; progressive fibrosis, often detectable before advanced LV dysfunction [24]	High arrhythmic-risk genotype where ICD consideration may precede severe LV dysfunction; risk escalates with conduction disease, NSVT, and fibrosis [22,26,27]
*FLNC (truncating variants)*	LV dilation with variable systolic dysfunction; often moderate impairment at presentation [35,36]	Low QRS voltages; inferolateral T-wave inversion; frequent PVCs/NSVT; VT/VF reported [29,30,31]	Extensive inferolateral or circumferential subepicardial LGE; fibrosis often disproportionate to remodeling [33,34]	ICD discussion may be warranted at relatively preserved/moderately reduced LVEF when VA and/or extensive LGE are present [37]
*RBM20*	Early LV dilation; frequently rapid decline in LVEF; aggressive DCM phenotype [46]	VAs common; NSVT and sustained VT may occur early [45,49]	Patchy/ring-like/diffuse mid-wall LGE; extensive fibrosis associated with arrhythmic events and HF progression [4,48]	ICD decisions often require integration of early VA (NSVT/VT), fibrosis burden, and disease trajectory rather than LVEF threshold alone [41,47]
*PLN (p.Arg14del)*	Mixed DCM/ACM phenotype; LV dilation with late systolic dysfunction; RV involvement may occur [52,55]	Low QRS voltages; atrial arrhythmias; NSVT; frequent ventricular ectopy [57,94]	Ring-like and/or inferolateral LGE; fibrosis may precede overt LV dysfunction [61,62]	Low-voltage ECG plus NSVT and/or LGE supports heightened concern and earlier ICD discussion in selected patients [63]
*Desmosomal genes*	RV dilation/dysfunction ± LV involvement; biventricular disease in advanced phases [65,66,73]	T-wave inversion (right and/or lateral leads); epsilon waves (subset); frequent ventricular ectopy and VT [70,76]	Fibrofatty replacement; subepicardial/transmural LGE involving RV and/or LV [77,98]	ICD decisions are frequently driven by arrhythmic phenotype and substrate (VT, syncope, high ectopy burden, and extensive scar), often independent of LVEF; early electrical instability may justify proactive strategy [69]

Abbreviations: NSVT, non-sustained ventricular tachycardia; LV, left ventricle; VA, ventricular arrhythmia; VT, ventricular tachycardia; VF, ventricular fibrillation; HF, heart failure; DCM, dilated cardiomyopathy; ACM, arrhythmogenic cardiomyopathy.

## Data Availability

No new data were created or analyzed in this study.
